# Histone H3K79 methyltransferase Dot1L is directly activated by thyroid hormone receptor during *Xenopus* metamorphosis

**DOI:** 10.1186/2045-3701-2-25

**Published:** 2012-07-16

**Authors:** Kazuo Matsuura, Kenta Fujimoto, Biswajit Das, Liezhen Fu, Christopher D Lu, Yun-Bo Shi

**Affiliations:** 1 Section on Molecular Morphogenesis, Program in Cellular Regulation and Metabolism (PCRM), Eunice Kennedy Shriver National Institute of Child Health and Human Development (NICHD), National Institutes of Health (NIH), 18 Library Dr, Bethesda, MD, 20892, USA; 2 Present address: Division of Gene Structure and Function, Research Center for Genomic Medicine, Saitama Medical University, 1397-1 Yamane, Hidaka-shi, Saitama, 350-1241, Japan; 3 Present address: Laboratory of Immunopathogenesis and Bioinformatics, Clinical Services Program, SAIC-Frederick, Inc, Frederick National Laboratory for Cancer Research, Frederick, MD, 21702, USA

**Keywords:** Dot1L, Intestinal stem cell development, Thyroid hormone receptor, Metamorphosis, *Xenopus laevis* and *tropicalis*, Histone methylation

## Abstract

**Background:**

Thyroid hormone (T3) is important for adult organ function and vertebrate development. Amphibian metamorphosis is totally dependent on T3 and offers a unique opportunity to study how T3 controls postembryonic development in vertebrates. Earlier studies have demonstrated that TR mediates the metamorphic effects of T3 in *Xenopus laevis*. Liganded TR recruits histone modifying coactivator complexes to target genes during metamorphosis. This leads to nucleosomal removal and histone modifications, including methylation of histone H3 lysine (K) 79, in the promoter regions, and the activation of T3-inducible genes.

**Results:**

We show that Dot1L, the only histone methyltransferase capable of methylating H3K79, is directly regulated by TR via binding to a T3 response element in the promoter region during metamorphosis in *Xenopus tropicalis,* a highly related species of *Xenopus laevis*. We further show that Dot1L expression in both the intestine and tail correlates with the transformation of the organs.

**Conclusions:**

Our findings suggest that TR activates Dot1L, which in turn participates in metamorphosis through a positive feedback to enhance H3K79 methylation and gene activation by liganded TR.

## Background

Thyroid hormone (T3) is important for proper development and normal physiology of many adult organs/tissues in vertebrates [1,2]. Sever T3 deficiency during human development leads to the formation of human cretins, who are short in stature and severely mentally retarded [3]. The most critical period of T3 action appears to be the several months around birth, the so-called postembryonic period, when T3 levels are high [1]. It has been difficult to study how T3 affects mammalian postembryonic development due to the lack of easily manipulatable models.

Metamorphosis in anurans such as *Xenopus laevis* or *tropicalis* mimics mammalian postembryonic development [1,4]. This process involves distinct changes in different organs and tissues [4,5]. The larval specific organs, such as the tail and gills, are totally resorbed during metamorphosis, while the adult specific ones, such as the limbs, develop de novo. Most of the organs/tissues are present in both tadpoles and frogs but are drastically remodeled during metamorphosis. For example, the animal intestine involves apoptotic degeneration of larval epithelial cells and concurrent de novo formation of adult stem cells to eventually develop the adult epithelium resembling that in mammals [6,7]. Interestingly, all such diverse changes during amphibian metamorphosis are totally dependent on T3 [5]. T3 can regulate transcription through T3 receptor (TR). TRs can form heterodimers with 9-cis retinoic acid receptors (RXRs) and repress or activate T3-inducible genes in the absence or presence of T3, respectively [2,8-10]. Recent studies have shown that TR appears to be both necessary and sufficient to mediate the metamorphic effects of T3 by regulating the transcription of T3 target gene [11-23]. In premetamorphic tadpoles, T3 levels are low and unliganded TR represses T3-inducible genes by recruiting histone deacetylase-containing corepressor complexes [16,24-26]. This helps to ensure proper premetamorphic growth and prevent premature metamorphosis [16,26]. When T3 becomes available, liganded TR recruits histone modifying coactivator complexes to these target genes, leading to histone modifications, chromatin remodeling and gene activation [18,19,22,27-31]. This results in metamorphic transformations of different organs.

TR is believed to regulate overlapping but distinct target genes in different organs to effect organ-specific metamorphosis. Toward identifying such target genes, we carried out a ChIP (chromatin immunoprecipitation)-on-chip analysis of the intestine by using a set of microarray chips covering a 8 kb region flanking each putative promoter of 17000 *Xenopus tropicalis* genes to look for genes bound by TR (unpublished observation). While the ChIP-on-chip data was very preliminary, it was of interest that one of the putative target genes thus identified corresponded to the *Xenopus tropicalis* homolog of the mammalian Dot1L gene.

Dot1L (Dot1-Like) is the homolog of yeast Dot1 gene, originally identified as a disruptor of telomeric silencing in *Saccharomyces cerevisiae*[32]. It belongs to the family of lysine methyltransferases (KMTs) [33-36]. Essentially all KMTs contain a SET (Su(var)3-9, Enhancer of Zeste (E(Z)), and Trithorax (trx)) domain. To date, Dot1L is the only known non-SET domain-containing KMT and is the only known KMT that possesses histone methyltransferase activity toward histone H3 lysine (K) 79 [33,34,37] Consistently, knockout of Dot1L in mice leads to a complete loss of H3K79 methylation [38].

H3K79 methylation is one of a large number of posttranslational modifications that occurs at the histone tails in eukaryotic cells [39]. In addition to K79, a number of other K and arginine (R) residues in both histone H3 and H4 can be methylated. These and other histone modifications are distributed in distinct patterns in the genome and variably associated with gene expression levels [22,40-49]. Among the well-characterized lysine methylation residues include K4, K9, K27, and K79 of histone H3. In general, H3K4 and H3K79 methylations correlate with high levels of transcription, while H3K9 and H3K27 methylations correlate with transcriptional repression. Of particular interest is the recent observation that H3K79 methylation levels are increased at TR target genes upon activation in *Xenopus tropicalis* intestine during natural and T3-induced metamorphosis [22]. This raises an interesting possibility that Dot1L may be transcriptionally activated by liganded TR and it in turn enhances chromatin remodeling and gene activation by liganded TR during development. Here we provide in vitro and in vivo evidence to show direct transcriptional activation of the Dot1L gene via the binding of TR to a T3 response element (TRE) in the Dot1L promoter in both the intestine and tail. We further show that Dot1L expression correlates with metamorphic changes in both organs, supporting a positive feedback role of Dot1L during metamorphosis.

## Results

### *Xenopus tropicalis* Dot1L is highly homologous to the mammalian Dot1L

**Figure 1:**
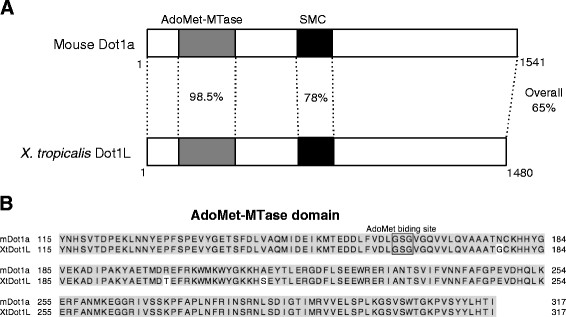
**Mouse and *Xenopus tropicalis *DOT1L proteins are highly conserved. (A)** Schematic alignment of mouse and *Xenopus tropicalis* DOT1L. The gray boxes represent conserved domains for S-adenosylmethionine (AdoMet or SAM)-dependent methyltransferases (AdoMet-MTases). The black boxes indicate structural maintenance of chromosome (SMC) domains. GenBank accession numbers: Mouse (m; *Mus musculus*) Dot1a (AAP42293) [50], *Xenopus tropicalis* (Xt) Dot1L (XP_002937984). Note that the N-terminal region is highly conserved between mouse and *Xenopus tropicalis* Dot1L due to the presence of the AdoMet-MTase and SMC domains. **(B)** The amino acid sequences of the nearly perfectly conserved AdoMet-MTase domain of mouse and *Xenopus* DOT1L. The AdoMet-binding site, which is essential for the methyltransferase activity, is boxed.

We obtained the full length coding region of *Xenopus tropicalis* Dot1L from GenBank and analyzed the domain structure of the predicted amino acid sequence by using NCBI’s Conserved Domain Database. Like yeast Dot1 and mammalian Dot1L, *Xenopus tropicalis* Dot1L has no SET domain (Figure 1A). The mouse and *Xenopus tropicalis* Dot1L proteins share 65% identity. The predicted AdoMet-MTase (S-adenosylmethionine (AdoMet or SAM)-dependent methyltransferase) domain is essentially identical between mouse and *Xenopus tropicalis* Dot1L and a conserved AdoMet binding site is also present in this region (Figure 1A and B). In addition, SMC (structural maintenance of chromosome) domain is also highly conserved, although its role in Dot1L remains unclear. It is possible that the SMC domain may play a role for heterochromatin formation since Dot1L is required for the formation of heterochromatin [38]. In any case, such a high degree of conservation indicates that *Xenopus tropicalis* Dot1L is a functional H3K79 methyltransferase.

### *Xenopus tropicalis* Dot1L is upregulated in both the intestine and tail during natural and T3-induced metamorphosis

**Figure 2:**
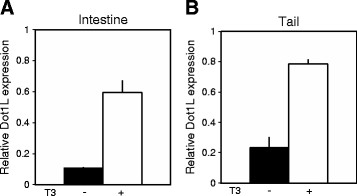
***Xenopus tropicalis *Dot1L is induced in the intestine and tail upon treatment of premetamorphic tadpoles with T3.** Stage 54 tadpoles were treated with 10 nM T3 for 2 days and total RNA was isolated from the intestine **(A)** and tail **(B)**. qRT-PCR was conducted to examine the expression of Dot1L mRNA. Dot1L expression was normalized to the expression of the control gene EF1α (elongation factor 1α). Error bars indicate s.e.m.

As indicated above, our preliminary ChIP-on-chip assay showed that Dot1L gene was bound by TR in the intestine of *Xenopus tropicalis* tadpoles (unpublished observation). This suggested that Dot1L might be regulated by T3 during metamorphosis. To investigate this possibility, we treated stage 54 premetamorphic tadpoles with 10 nM T3 for 2 days. Dot1L expression was analyzed on total RNA isolated from the intestine and tail by qRT-PCR. Figure 2 showed that T3 strongly induced the expression of Dot1L in both the intestine and tail, even though these two organs undergo vastly different changes during metamorphosis. These results suggest that Dot1L may play a role in diverse tissues during metamorphosis.

**Figure 3:**
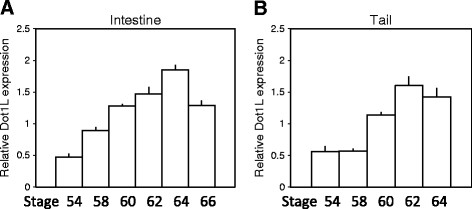
**Upregulation of *Xenopus tropicalis *Dot1L expression in the intestine and tail during natural metamorphosis.** Total RNA was isolated from the intestine **(A)** and tail **(B)** at the indicated developmental stages from premetamorphosis to the end of metamorphosis (stage 66). The expression of Dot1L mRNA was determined by qRT-PCR as in Figure 2. Error bars indicate s.e.m. Note that there were no tail samples at stage 66 **(B)** as the tail is completely resorbed by stage 66.

To investigate whether Dot1L expression is also regulated by T3 during natural metamorphosis, we analyzed its expression by qRT-PCR in the intestine and tail at different stages from premetamorphic (stage 54), metamorphic climax (stages 58-64), to the end of metamorphosis (stage 66). The results in Figure 3 showed that Dot1L expression was upregulated in the intestine and tail during metamorphosis correlating with the rising concentration of plasma T3 during metamorphosis. Thus, Dot1L is upregulated by T3 and likely participates in the metamorphic transformations of different organs.

### TR/RXR heterodimer can bind to two putative TREs in the Dot1L gene promoter region in vitro

**Figure 4:**
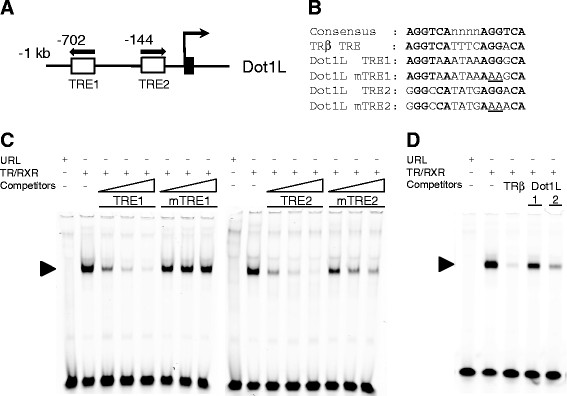
**TR binds to the putative TREs in Dot1L gene in vitro. (A)** Schematics diagram of the putative TREs in *Xenopus tropicalis* Dot1L gene. The TREs are shown as white boxes with arrows indicating the orientation of TREs. The black box shows exon. **(B)** The sequences of wild type and mutant Dot1L TRE1 and TRE2 used in the gel shift assay in comparison to the consensus TRE and the TRE of *Xenopus laevis* TRβA gene. Bold letters show conserved nucleotides and the mutated nucleotides are underlined. **(C)** Both Dot1L TRE1 and TRE2 compete against the TRE of *Xenopus laevis* TRβA for binding to TR/RXR heterodimers. The labeled *Xenopus laevis* TRβA promoter TRE was mixed with *in vitro* translated TR/RXR heterodimers in the presence or absence of 4×, 20×, or 100× unlabeled wild type or mutant Dot1L TRE1 or TRE2 as indicated. The reaction mixture was analyzed by gel retardation assay. Arrowhead indicates the TR/RXR-TRE complex. Note that both wild-type Dot1L TREs competed while the mutations reduced or abolished their ability to compete for binding to TR/RXR. **(D)** Dot1L TRE2 has higher affinity than TRE1 for TR/RXR heterodimer in vitro. Gel mobility shift assay was done as in **(C)** except with 20× unlabeled *Xenopus laevis* TRβA TRE, Dot1L TRE1, and Dot1L TRE2. Note that *Xenopus laevis* TRβA TRE competed most effectively, followed by Dot1L TRE2, while Dot1L TRE1 was least effective.

As our preliminary data showed that TR was associated with the Dot1L gene promoter in the tadpole intestine, we carried out a bioinformatics analysis on the promoter region and found two putative TREs near the predicted transcription start site (Figure 4A). Since TR/RXR heterodimers bind to TREs of T3 induced genes, we investigated if TR/RXR heterodimers could bind to the TREs. We carried out in vitro gel mobility shift assays by using IR700-labeled TRE of *Xenopus laevis* TRβA gene (Note that there are two duplicated TRβ genes in *Xenopus laevis*, TRβA and TRβB that are similarly regulated [51]), a well characterized TRE consisting of two near perfect direct repeats of AGGTCA separated by 4 bp [52,53], and in vitro translated TR and RXR in the presence or absence of unlabeled competitor TREs from the *Xenopus tropicalis* Dot1L gene. To determine the binding specificity, we also generated mutated TRE1 and TRE2 (Figure 4B) for the competition assay. Both unlabeled TRE1 and TRE2 were able to compete against the labeled TRβA TRE for binding to TR/RXR and mutating TRE1 and TRE2 reduced this competition (Figure 4C).

To compare the relative affinity of the TREs, we carried out competition assays with equal amount of unlabeled TRE1, TRE2, or TRβA TRE. The results in Figure 4D indicated that the TRβA TRE had the strongest competition, followed by TRE2, with TRE1 being the weakest competitor. Thus, both TREs could bind to TR/RXR with TRE2 having a higher affinity in vitro.

### TR/RXR regulates the Dot1L promoter in vivo via TRE2

**Figure 5:**
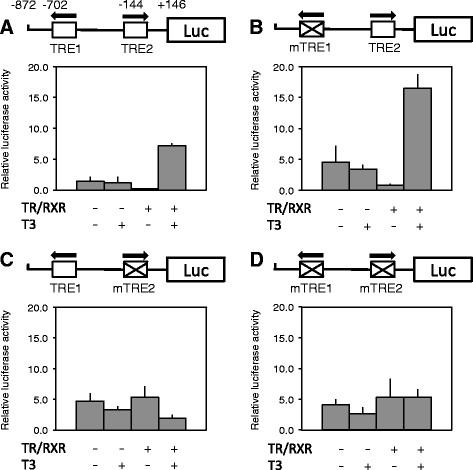
**The activation of *Xenopus tropicalis *Dot1L promoter by liganded TR in vivo is dependent on TRE2.** On the top of each panel shows a schematic diagram of the promoter construct with or without mutations in TRE1 (mTRE1) and/or TRE2 (mTRE2). The lower portion shows the promoter activity in the frog oocytes. For the transcription assay, wild type or mutant promoter construct was co-injected with the control *Renilla* luciferase construct phRG-tk into the nuclei of the oocytes with or without prior cytoplasmic injection of mRNAs for *Xenopus tropicalis* TRα and RXRβ. The oocytes were incubated at 18°C overnight in the presence or absence of 100 nM T3 and then used for dual luciferase assays. The relative activities of the firefly luciferase to *Renilla* luciferase were plotted. Error bars indicate s.e.m. Note that mutation of TRE1 alone had no effect on the regulation of the promoter by TR/RXR both in the presence or absence of T3 while mutating of TRE2 alone or together with TRE1 abolished the regulation by TR/RXR, suggesting that TRE2 is the functional TRE

To determine whether TRE1 and TRE2 can mediate the transcriptional regulation of the Dot1L gene by T3 in vivo, we used a reconstituted *Xenopus laevis* oocyte transcription system, which allows the analysis of the promoter in the context of chromatin in vivo [54]. First, we microinjected mRNAs encoding TR and RXR into *Xenopus* oocyte cytoplasm to allow the synthesis of the proteins. Then, we microinjected the reporter plasmid containing the Dot1L promoter driving the firefly luciferase together with an internal control plasmid phRG-tk expressing *Renilla* luciferase into oocyte nucleus. After overnight incubation of the oocytes in the presence or absence of T3, they were harvested for the luciferase assay with the ratio of firefly luciferase to *Renilla* luciferase activities as a measure of the promoter activity. As shown in Figure 5A, the wild type Dot1L promoter was not affected by T3 treatment of oocytes without pre-injection of TR/RXR mRNAs. When TR/RXR were expressed, the promoter was repressed in the absence of added T3. When T3 was present, the promoter was strongly activated (Figure 5A), demonstrating that the Dot1L promoter could be regulated by TR/RXR in vivo.

To determine the roles of the individual TREs in mediating the effect of TR/RXR, each was mutated individually or together and the resulting promoters were analyzed in the oocyte. Mutating TRE1 had little effect on the regulation of the promoter by TR/RXR in the presence or absence of T3 (Figure 5B). On the other hand, mutating TRE2 alone (Figure 5C) or together with TRE1 (Figure 5D) abolished both the repression by unliganded TR/RXR and the activation by TR/RXR in the presence of T3. Thus, TRE2 is mainly responsible for mediating the transcriptional effects of TR/RXR in the presence or absence of T3 in vivo, consistent with the higher affinity of TRE2 for TR/RXR in vitro.

### TR is bound to the Dot1L TRE2 in both intestine and tail of premetamorphic tadpoles

**Figure 6:**
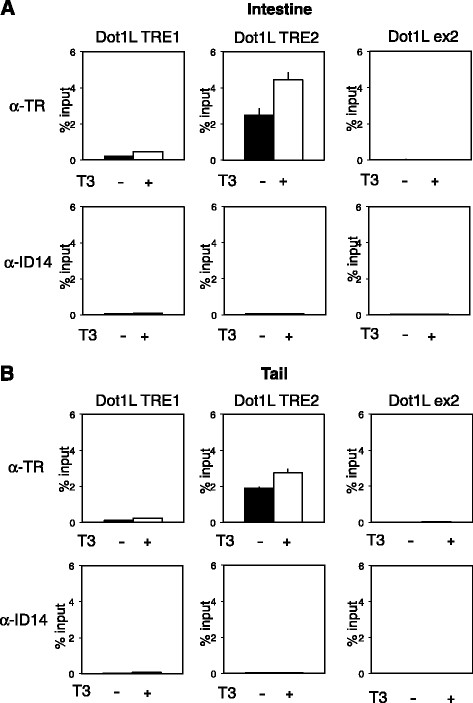
**TR binds to the TRE2 in Dot1L promoter in the intestine and tail during T3-induced metamorphosis**. Stage 54 tadpoles were treated with or without T3 for 2 days. The intestine **(A)** or tail **(B)** was isolated for ChIP assay with a polyclonal antibody against TR (top) or Id14 (bottom), a negative control for antibody specificity. The immunoprecipitated DNA was analyzed by qPCR for the presence of the TRE regions of Dot1L promoter. A region of exon 2 (ex2) of Dot1L gene was analyzed as a negative control for binding specificity. Note that strong binding of TR to TRE2 in Dot1L gene was observed in the absence of T3 in premetamorphic tadpoles and that this binding was increased slightly upon T3 treatment in both organs. TR signal at the TRE1 was very low in both organs with or without T3 treatment, consistent with the results from transcription and *in vitro* TR binding assays. Only background signals were observed with the anti-ID14 antibody. Error bars indicate s.e.m

Our preliminary ChIP-on-chip assay suggested that the Dot1L promoter region was bound by TR in the intestine of premetamorphic *Xenopus tropicalis* tadpoles*.* To confirm the binding of TR to the TRE regions of Dot1L gene in vivo, we carried out ChIP assays with a polyclonal anti-TR antibody on the intestine of stage 54 premetamorphic tadpoles treated with or without T3. As a control for antibody specificity, we carried out parallel ChIP assay with a polyclonal antibody against ID14, an extracellular matrix protein [55]. Figure 6A showed that even in the absence of T3 treatment, ChIP signal for TR was very strong at the TRE2 region and the signal was elevated further upon T3 treatment. On the other hand, there was very little ChIP signal for TR at the TRE1 region of Dot1L gene. It is worth noting that the low levels of the ChIP signal associated with TRE1 were still significantly above the background signal observed with the ID14 antibody. In addition, a negative control for binding specificity by analyzing the Dot1L exon 2 region far away from the promoter also yielded very low background signals (Figure 6A). These results suggest that the signal at the TRE1 was specific for TR. On the other hand, as the DNA generated by sonication during the ChIP assay could be as long as 1 kb (typically averaging around 500 bp), we could not rule out the possibility that the signal observed at TRE1 was due to TR binding at TRE2. This could occur if the TRE1 region was present in some large fragments of DNA immunoprecipitated due to TR binding to the TRE2. In any case, our results thus confirmed our preliminary ChIP-on-chip findings and further suggest that TR bind preferentially, if not exclusively, to the TRE2 of the Dot1L promoter to regulate its transcription in the intestine during metamorphosis.

As Dot1L was also upregulated in the tail during metamorphosis, we analyzed TR binding to the TREs in the tail of the same tadpoles as studied above for the intestine. Essentially identical results were obtained (Figure 6B). That is, TR also bound predominantly to the TRE2 in the tail of premetamorphic tadpoles treated with or without T3 for 2 days. Thus, TRE2 likely mediates the induction of Dot1L by T3 in different organs during metamorphosis.

## Discussion/conclusion

TR can activate and repress T3-inducible genes in the presence and absence of T3, respectively. TR functions by recruiting cofactor complexes, many of which can modify histones at the target genes. Extensive genome-wide analyses in cell cultures have identified a number of histone marks that are associated with active or repressed genes, respectively [41-49]. However, the roles of such histone modifications and the corresponding enzymes in gene regulation by TR, especially during vertebrate development, largely remain to be investigated. Amphibian metamorphosis offers a unique opportunity to study the in vivo function and mechanisms of TR during development because of its total dependence on T3 and ability to be easily manipulated. Our earlier studies have shown that transcriptional activation of target genes during *X. tropicalis* metamorphosis is accompanied by nucleosome eviction and histone modifications, include H3K79 trimethylation [22]. Here we have demonstrated that the only known histone H3K79 methyltransferase Dot1L is transcriptionally regulated by TR during metamorphosis.

We initially identified Dot1L as a likely TR target from a preliminary ChIP-on-chip analysis, which showed TR association with the putative promoter region of the gene in premetamorphic tadpole intestine (unpublished observation). Our bioinformatics analysis revealed two putative TREs in this promoter region. Furthermore, both TREs were capable of binding to TR/RXR heterodimers in vitro with TRE2 having a higher affinity. More importantly, we demonstrated that in the frog oocyte transcription system in vivo, TRE2 but not TRE1 was essential for TR/RXR to regulate the Dot1L promoter in a T3 dependent manner. Further evidence supporting a critical role of TRE2 in Dot1L expression came from in vivo ChIP assay. In both animal tail and intestine, TR binding was much stronger to TRE2 than to TRE1. As indicated above, given the limitation of the ChIP assay, it is possible that the weak TR ChIP signal at TRE1 was a result of TR binding at the TRE2 and TRE2 is the only TRE bound by TR during metamorphosis in the intestine and tail. In any case, our findings together indicate that Dot1L gene is a direct T3 target gene that is regulated via binding of the TR predominantly to TRE2.

Our expression analyses indicated that Dot1L is upregulated during metamorphosis in both intestine and tail, temporally correlating with their metamorphic transformations. The tail and intestine undergo vastly different changes during metamorphosis, total resorption vs. remodeling involving both larval cell death and adult cell development. The upregulation of Dot1L in both organs suggest that Dot1L participates in the metamorphosis in different organs/tissues.

How Dot1L is involved in metamorphosis remains to be investigated. Interestingly, we have recently shown that H3K79 methylation levels are dramatically upregulated at two well characterized TR target genes, the TRβ and TH/bZip genes, in the intestine of *Xenopus tropicalis* tadpoles during natural or T3-induced metamorphosis [22]. Given the fact that Dot1L is the only known H3K79 methyltransferase in the vertebrate genome [33,38], it is very likely that the increase in H3K79 trimethylation at the TR target genes is mediated by Dotl1L. Therefore, it is tempting to speculate that Dot1L is directly induced by liganded TR early during metamorphosis in different organs such that the increased levels of Dot1L in turn feed back positively to increase H3K79 methylation induced by TR. This further enhances gene regulation by TR during metamorphosis. Whether and how Dot1L participates in histone modification and gene regulation by TR in vivo will be an important subject for future studies. In addition, it would be interesting to determine the role of Dot1L in the transformation of different organs during metamorphosis.

## Materials and methods

### Experimental animals

*Xenopus tropicalis* tadpoles and *Xenopus laevis* frogs were purchased from Nasco (Fort Atkinson, MI). Tadpoles were staged according to Nieuwkoop and Faber [56]. When indicated, stage 54 tadpoles were treated with 10 nM T3 for 2 days at 22 °C. All animal procedures were done as approved by NICHD Animal Use and Care Committee.

### Quantitative RT-PCR

Total RNA was isolated from tadpole intestine and tail at indicated stages. cDNA was synthesized from 2.5 μg of total RNA by using the High Capacity cDNA Archive kit (Applied Biosystems) in 50 μl according to the manufacturer’s instructions. qRT-PCR was carried out by using SYBR Green PCR Master Mix (Applied Biosystems). EF1α (elongation factor 1α) was used as the normalization control as described previously [57]. The primers forward 5’-TACACACTGGAGCGTGGAGA-3’ and reverse 5’- GGTCAACCTCAGGACCAAAG -3’ were used for quantifying Dot1L mRNA levels.

### Generation of promoter constructs

The Dot1L promoter region was PCR-amplified from *Xenopus tropicalis* genomic DNA with a primer pair 5’-CCCCGGTACCGTTTCCTAGGCGGCGGAGGATGCCC-3’ (bearing KpnI site at its 5’-end) and 5’-CCCCCTCGAGCATACTAATCCAACTCCGCCGGCGGTG-3’ (bearing XhoI site at its 5’-end). The PCR product was cloned into KpnI and XhoI sites of pGL4.10 firefly luciferase vector (Promega). The mutant promoters harboring mutant TREs were generated from the wild type construct using QuickChange Lightning Site-Direct Mutagenesis Kit (Agilent/Stratagene) according to manufacturer’s instructions. The primers used for mutagenesis were: KF385 5’-AGCATTGTTAGTAATCCGCTGCTTTTTATTTATTTCACTGGCACCATCACCTTGCG-3' (the mutated nucleotides are underlined) to generate mTRE1, KF386 5’-CCCTCAGACTTCAGGCTAAGGAACCATATGAAAACACCCCGGGATTATTTATT-3’ to generate mTRE2. Both KF385 and KF386 were used to generate the construct mTRE1 mTRE2 where both TREs were mutated. The mutated constructs were confirmed by DNA sequencing.

### Bioinformatics identification of TREs

The computational analysis program NHR-Scan (http://asp.ii.uib.no:8090/cgi-bin/NHR-scan/nhr_scan.cgi) [58] was used to search for putative TREs.

### Transcription assay in the *Xenopus laevis* oocyte system

Oocyte transcription assay was performed as described [23,54]. Briefly, the plasmids containing *Xenopus tropicalis* TRα and RXRβ were linearized and transcribed *in vitro* using a T7 kit (Life technologies/Ambion, Grand Island, NY). The cytoplasm of stage VI oocytes from *Xenopus laevis* was injected with 46 pg/oocyte of the TR and RXR mRNAs. Two hour later, the firefly luciferase reporter under the control of the wild type or mutant promoter of *Xenopus tropicalis* Dot1L gene (33 pg/oocyte) and the internal control *Renilla* reporter phRG-TK (33 pg/oocyte) were co-injected into the nucleus. After incubation at 18°C overnight in the presence or absence of 100 nM T3, the injected oocytes were prepared for luciferase assay using the Dual-Luciferase-Reporter Assay system according to the manufacture’s protocol (Promega). Six oocytes per sample were lysed in 90 μl of 1× lysis buffer (Promega), and 10 μl of lysate was used for luciferase assays. Three independent samples were done for each injection at the same time. The relative expression of firefly luciferase to *Renilla* luciferase was determined. Each data point represents the average of three groups. The data shown here were a representative of a few independent experiments with similar results.

### Chromatin immunoprecipitation (ChIP) assay

ChIP assay on tadpole intestine and tail was done as described previously [22] with anti-TR (new PB) antibody [23]. As a negative control, a polyclonal antibody against Id14, an extracellular protein [55], was also used. All treatment and control groups had three replicas, and each replica consisted of six to eight tadpoles. All ChIP experiments were done twice with similar results.

The immunoprecipitated DNA was analyzed by quantitative PCR using the following primers: 5’-ACGCGTATCGGACCCACGCAAGGTG-3’ and 5’-AGACTTCGACAGCCTACGTGAGCAGCA-3’ for Dot1L TRE1 region, 5’-TGTGGCCTACGAAGCGACCGCCTTC-3’ and 5’-TCAGGCTAAGGGGCCATATGAGGACACCC-3’ for Dot1L TRE2 region, 5’-GATAAACACCATGATGCTGCAC-3’ and 5’-TACACCACTCCGTGAGTAGCAC-3’ for Dot1L exon 2.

### Gel mobility shift assay

Gel mobility shift assay was performed as described [57]. Briefly, *Xenopus laevis* TR and RXR proteins were made using TNT SP6 Quick Coupled transcription/translation System (Promega, Madison, WI). They were mixed with infrared dye IR700 (LI-COR, Lincoln, NE)-labeled TRE of the *Xenopus laevis* TRβA gene [52] in the in vitro binding reaction in the presence or absence of unlabeled competitors made of wild type or mutant TREs from *Xenopus tropicalis* Dot1L gene. The sequences of sense strand of oligonucleotides for the Dot1L TREs were as follows: 5’-AATCCGC**TGCCCT**TTAT**TTACCT**CACTGGC-3’ for Dot1L TRE1 and 5’-AATCCGC**TGCTTT**TTAT**TTACCT**CACTGGC-3’ for mutant Dot1L TRE1, and 5’-GGCTAAG**GGGCCA**TATG**AGGACA**CCCGCGG-3’ for Dot1L TRE2 and 5’-GGCTAAG**GGGCCA**TATG**AAAACA**CCCGCGG-3’ for mutant Dot1L TRE2 (Bold letters indicates the TRE half sites, and the mutated nucleotides are underlined). Each binding reaction included one hundred fmol (1μl) of IR700-labeled probe, 1 μl of each TR and RXR in vitro translation mixture, and 1μl of the wild-type or mutant TRE oligonucleotides at 400 fmol/μl, 2 pmol/μl, or 10 pmol/μl to obtain 4×, 20×, 100× unlabeled competitor oligonucleotides, in a total volume of 20 μl. The mixtures were incubated at room temperature for 20 min, electrophoresed on a 6% DNA retardation gel (Invitrogen), and then scanned using Odyssey Infrared scanner (LI-COR, Lincoln, NE).

## Abbreviations

Dot1L, Disruptor of telomeric silencing 1-like; T3, Thyroid hormone; TR, T3 receptor; RXR, 9-cis retinoic acid receptor; TRE, T3 response element; EF1α, Elongation factor 1α; qRT-PCR, Quantitative reverse-transcription polymerase chain reaction; ChIP, Chromatin immunoprecipitation; KMT, Lysine methyltransferase; AdoMet or SAM, S-adenosylmethionine; AdoMet-MTase, AdoMet-dependent methyltransferase.

## Competing interests

None.

## Authors’ contributions

KM, KF, BD, LF and CDL designed and carried out experiments and interpreted the findings; KF prepared the manuscript; YBS supervised the study and prepared the manuscript. All approved the manuscript.
